# Supplementary material for: Large structural variants in KOLF2.1J are unlikely to compromise neurological disease modelling
Source: bioRxiv. 2024 Jan 29:2024.01.29.577739. Preprint. [Version 1] doi: 10.1101/2024.01.29.577739 (PMC10862770; doi:10.1101/2024.01.29.577739)
Supplement: Supplement 1 [file NIHPP2024.01.29.577739v1-supplement-1.pdf]

bioRxiv preprint doi: https://doi.org/10.1101/2024.01.29.577739; this version posted January 29, 2024. The copyright holder for this preprint (which was not certified by peer review) is the author/funder, who has granted bioRxiv a license to display the preprint in perpetuity. It is made available under aCC-BY 4.0 International license.

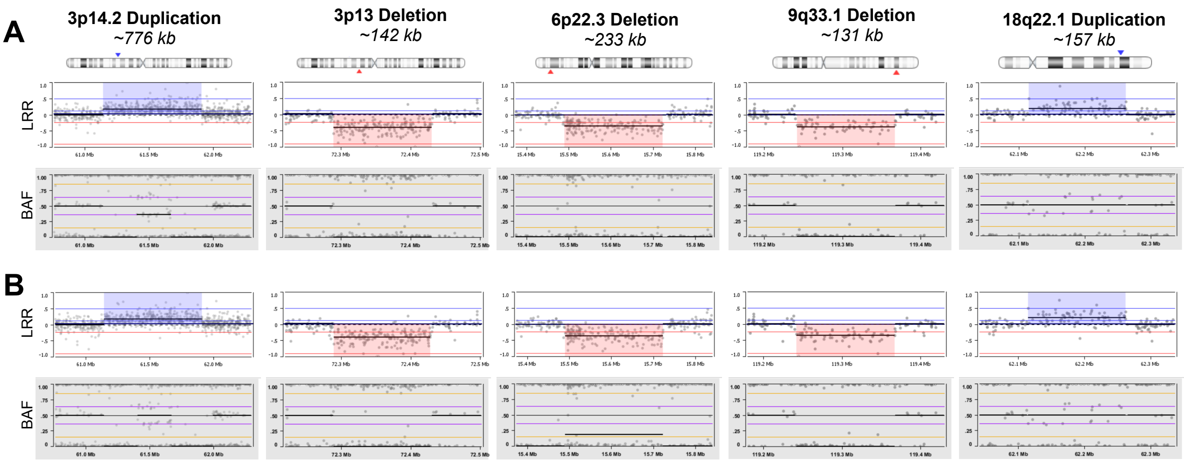

C

CNV region	CNV type	Affected gene	pHaplo ≥ 0.86?	pTriplo ≥ 0.94?	GnomAD LOEUF ≤ 0.35?	ClinGen dosage sensitivity?	Mouse +/- phenotype?
Chr3p14	duplication	*FHIT*	n/a	No, 0.336	n/a	No evidence for triplosensitivity	n/a
Chr3p14	duplication	*PTPRB*	n/a	No, 0.821	n/a	No data	n/a
Chr6p22	deletion	*DTNBP1*	No, 0.769	n/a	No, 0.984	No data	none reported[10]
Chr6p22	deletion	*JARID2*	Yes, 0.997	n/a	Yes, 0.188	No evidence for haploinsufficiency	none reported[7]
Chr9q33	deletion	*ASTN2*	Yes, 0.884	n/a	Yes, 0.248	Little evidence for haploinsufficiency	none reported[8]

Figure S1. Copy number variants in KOLF2.1J and parental KOLF2_C1 iPSC lines. **A,B)** Plots of structural variants from DNA microarray data called by Log R Ratio (LRR) and B Allele Frequency (BAF) in KOLF2.1J (A) and KOLF2_C1 (B) showing two duplicated regions and three deleted regions visualised by VIA software v7.0 (Bionano Genomics). **C)** While bioinformatic tools predict potential pathogenicity of heterozygous variants in Chr6p22 and Chr9p33 deletions, manual annotation in ClinGen and heterozygous mouse models do not support this interpretation.
